# Effect of Peptide-Conjugated Near-Infrared Fluorescent Quantum Dots (NIRF-QDs) on the Invasion and Metastasis of Human Tongue Squamous Cell Carcinoma Cell Line Tca8113 *in Vitro*

**DOI:** 10.3390/ijms10104418

**Published:** 2009-11-20

**Authors:** Kai Yang, Zhigang Li, Yuan Cao, Xiuli Yu, Jie Mei

**Affiliations:** Department of Oral and Maxillofacial Surgery, the First Affiliated Hospital, Chongqing Medical University, Chongqing 400016, China; E-Mails: jim19830105@163.com (Z.L.); caoyuan219@126.com (Y.C.); yuxiuli876@sina.com (X.Y.); kathydl@tom.com (J.M.)

**Keywords:** peptide, near-infrared fluorescence, quantum dots, tumor, adherence, invasion, chemotaxis

## Abstract

In this study we investigated the effect of near-infrared fluorescent quantum dots (NIRF-QDs, QTracker) on the proliferation, adherence, invasion and chemotaxis of human tongue squamous cell carcinoma cell line Tca8113 *in vitro*. Cell proliferation and colony formation rate were determined by using a hemocytometer and culture plate. A transwell chamber assay was used to determine the cell invasion, adherence and chemotaxis. The results showed that there was no significant difference between the results of Tca8113 cells labeled with NIRF-QD800 and those of unlabeled Tca8113 cells, suggesting that the proliferation, invasion, adherence and chemotaxis of Tca8113 cells were not affected by NIRF-QD800. These results provide a basis for the further utilization of NIRF-QDs in non-invasive imaging and tracking of tumor cells *in vivo*.

## 1. Introduction

Dynamic and *in situ* monitoring the genesis, development, infiltration, metastasis and distribution of cancer cells *in vivo* are the keys for the investigation of tumor occurrence, advances, early diagnosis and treatment. The primary technique to solve these problems is to develop an optical marker that is easily detectable and suitable for *in vivo* applications [1–3].

Recently developed semiconductor quantum dots (QDs) have several advantages over the conventional organic (*e.g.*, rhodamine, FITC, Cy3 and Cy5) and fluorescent protein probes. For example, QDs have a high photoluminescence quantum yield, high intense fluorescence, excellent photostability and high resistance to photobleaching and photodegradation [4,5]. In addition, their absorption spectra are wide, continuous and can be excited at any wavelength shorter than their emission peak. Their emission spectra are narrow, symmetrical and the effective Stokes shifts are large. QDs of different semiconductors or the same semiconductor with different particle sizes can emit different colors simultaneously with a single excitation light source, which makes it easy to recognize and distinguish different kinds of cells labeled with different sizes of QDs. The luminescence feature of QDs displays strict size effect: all kinds of spectra from UV to infrared wavelength or from blue to red wavelength can be obtained by changing the size of QDs [6]. Conventional fluorescent probes do not have these intrinsic luminescent characteristics of QDs. Therefore, live cell labeled with QDs makes it possible for long-term or simultaneous observation of multiple cell interactions [7]. Excellent luminescent characteristics of QDs have shown tremendous prospects in the non-invasive, *in situ* studies of the tumor genesis, development, early diagnosis and transportation of drugs *in vivo* [8,9]. The recently developed QDs with an infrared emission wavelength between 700 and 900 nm has a powerful ability to penetrate the human tissues and in the mean time it can avoid the interference of tissue autofluorescence (400–600 nm), which is particularly suitable for the non-invasive medical imaging.

Currently, QDs have been used in non-invasive imaging of tumor cells [7,10,11], detection of sentinel lymph nodes [12] and targeting imaging of tumor neovasculature in the live subjects through non-specific endocytosis of QDs or specific interaction between antibody-, ligand-, peptide-conjugated fluorescent probes and targeted molecule in the tissues. Studies suggested that labeling of tumor cells with bioconjugated QDs was not toxic and did not affect the biological behavior of tumor cells *e.g.*, growth and differentiation [2,7]. Some studies used QDs labeled cancer cells for the non-invasive imaging of cell invasion and metastasis [7,10–12]. However, the key issue of whether or not QDs-labeled tumor cells affect the invasion and metastasis ability has not been reported. The key issue that needs to be resolved is if the results obtained from the non-invasive visualization of QDs-labeled tumors are scientific and if the results reflect the actual tumor cell infiltration and metastasis *in vivo*.

Ninety percent of the malignant tumor in the oral and maxillofacial regions is squamous cell carcinoma. Tongue squamous cell carcinoma is one of the most common oral squamous cell carcinomas, with only 50% survival rate at five years and thus investigation on this tumor is of high significance. Studies have shown that membrane-penetrating peptide-conjugated QDs can label the cells effectively and rapidly [13]. In this study, we used peptide-conjugated near-infrared fluorescent QD (Qtracker^TM^ QD800) with a maximal emission of 800 nm to label human tongue squamous cell carcinoma cell line (Tca8113) through endocytosis. We then observed the proliferation, adherence and chemotaxis of the QD800-labeled Tca8003 cells. These results provided theoretical bases to reveal if the visualized tumor cells labeled by peptide-conjugated near-infrared fluorescent QDs reflected the actual genesis, development, infiltration and metastasis of tumors *in vivo*.

## 2. Results and Discussion

### 2.1. Results

#### 2.1.1. Labeling of Tca8113 Cells with QDs

**Figure 1.**
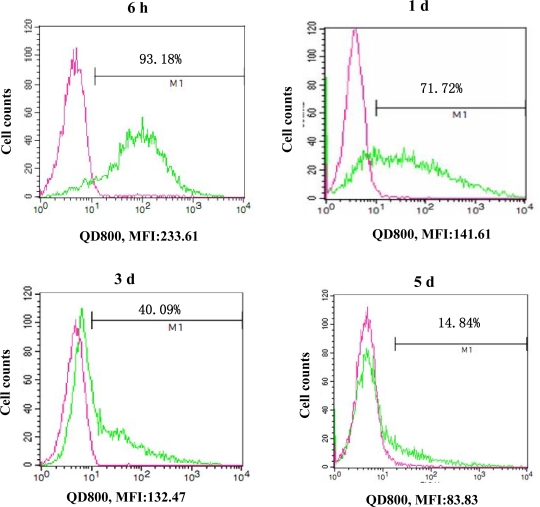

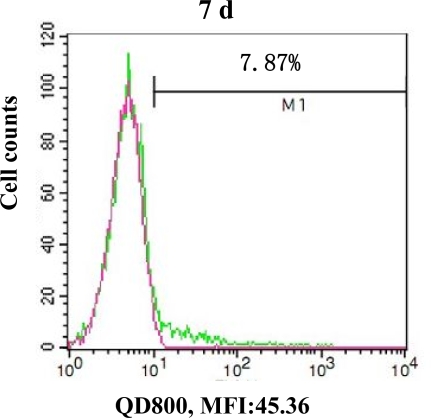
Qtracker intracellular QD800 delivery quantified by flow cytometry. Flow cytometry detection of QD800 labeling of Tca8113 cells on 6 h, 1 d, 3 d, 5 d, and 7 d. Red line represented unlabeled cells as control; green line represented cells labeled with QD800.

**Figure 2.**
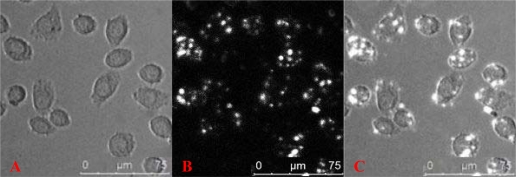
Fluorescent images of cells labeled with QD800 on day 1 post labeling. (A) Bright-field, (B) QD800 fluorescence image, (C) overlay image.

The labeling rate of Tca8113 cells after 6 h, 1 d, 3 d, 5 d and 7 d was 93.18%, 71.72%, 40.09%, 14.84% and 7.87%, respectively, as detected by flow cytometry (Figure 1). The average fluorescent intensity was 233.61, 141.61, 132.47, 83.83 and 45.36, respectively. After 1 day of labeling, a large amount of QD800 was distributed in the cytoplasm, as detected by a laser confocal microscope (Figure 2).

#### 2.1.2. Effect of QDs on the Proliferation of Tca8113 Cells

**Figure 3.**
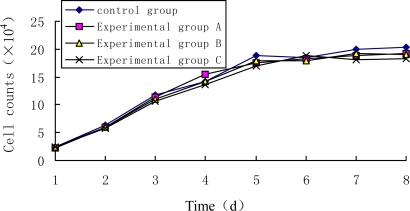
Growth curve of Tca8113 cell treated or untreated with QD800. (i) growth curve of unlabeled Tca8113 cells; (ii) growth curve of Tca8113 cells labeled with 5 nM QD800; (iii) growth curve of Tca8113 cells labeled with 10 nM QD800; (iv) growth curve of Tca8113 cells labeled with 15 nM QD800.

The growth curves of unlabeled Tca8113 or Tca8113 cells labeled with different concentrations of QDs are shown in Figure 3. The results indicated that there was no significant difference between the growth curved of QDs-labeled and unlabeled Tca8113 cells, indicating that the presence of QD800 does not affect the growth of Tca8113 cells.

#### 2.1.3. Effect of QDs on the Tca8113 Cell Clone Formation

**Figure 4.**
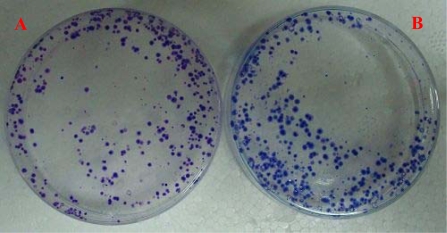
The clone formation efficiency of Tca8113 cell treated or untreated with QD800 after 21 d of culture. Panel A indicated the unlabeled Tca8113 cells and panel B indicated Tca8113 labeled with QD800.

The clone formation efficiency of Tca8113/QD800 and Tca8113 cells were (64.7 ± 5.4)% and (66.2 ± 3.1)%, respectively, after 21 d of culture (Figure 4). Statistical analysis showed that there was no significant difference between the clonal formation rate of QDs-labeled cells and that of unlabeled cells, indicating that labeling of Tca8113 cells with QD800 does not affect the clone forming ability.

#### 2.1.4. Effect of QDs on the Tca8113 Cell Adhesion

The adhesion rate of Tca8113/QD800 and Tca8113 cells at different time points was shown in Table 1. There was no significant difference between the adhesion rate of Tca8113/QD800 and that of Tca8113 cells at each time point (Table 1), indicating that labeling of Tca8113 cells with QD800 does not affect the cell adhesion.

#### 2.1.5. Effect of QDs on the Tca8113 Cell Invasion

Matrigel poured on the surface of porous filter membrane formed a structure similar to the natural basal membrane. The ability of the cells to penetrate matrigel reflected its invasive capability. The results showed that the average number of Tca8113/QD800 and Tca8113 cells that penetrated the membrane was 70.3 ± 2.7 and 72.6 ± 2.4, respectively. Statistical analysis showed that there was no significant difference between the cell invasion ability of QDs-labeled cells and that of unlabeled cells (*P* > 0.05), indicating that labeling of Tca8113 cells with QD800 does not affect their invasion ability.

#### 2.1.6. Effect of QDs on the Tca8113 Cell Chemotaxis

The average number of Tca8113/QD800 and Tca8113 cells that penetrated the membrane without matrigel was 79.9 ± 4.3 and 83.6 ± 3.5, respectively. Statistical analysis showed that there was no significant difference between the number of translocated Tca8113/QD800 cells and that of Tca8113 cells (*P* > 0.05), indicating that labeling of Tca8113 cells with QD800 does not affect their chemotaxis.

### 2.2. Discussion

Developing non-invasive and visual method for monitoring tumor cells *in vivo* is important for studies on the genesis, early diagnosis and treatment of tumors. Compared to conventional organic fluorescent dyes or fluorescent proteins, QDs have the excellent luminescent features of stability and high yield of fluorescent quantum. QDs technology has displayed its promising prospects in long-term and non-invasive monitoring and tracking tumor cells *in vivo* [7]. Currently, the approaches that are used for QDs labeling of live cells include liposome-mediated transduction, non-specific internalization, micro-injection, electroporation and monoclonal or peptide-mediated transduction [14–20]. Previous studies have demonstrated that liposome-mediated QDs internalization displayed vesicle-like compartmental aggregation within the cells, which makes it difficult to control the concentration of QDs [14]. Non-specific internalization is only suitable for the labeling of strong phagocytic cells [2]. Micro-injection is also suitable for the labeling of small amount of cells [15,16]. Electroporation causes damages on the cells, leading to the death of some cells [14,17]. Recent studies demonstrate that cell-penetrating peptide conjugated QDs are suitable for rapid and efficient labeling of various types of cells. More importantly, these conjugated QDs do not have damaging effect on the cells [18–20]. Lin *et al*. utilized near-infrared fluorescent quantum dots (Qtracker^TM^ QD800) to label mouse embryonic cells and demonstrated that QD800 not only had excellent *in vivo* imaging ability, but also did not affect the viability and differentiation of embryonic stem cells. These results provided fundamental bases for further studies on the imaging and tracking of stem cells by quantum dot labeling. Although studies have been reported with regard to the QD800 imaging of tumor cells to investigate the invasion and metastasis *in vivo* [12,13], the effect of QD800 on the invasion and metastasis has not been reported. We showed here that co-culture of QD800 with Tca8113 cells resulted in the rapid entrance of QD800 into the cells and the labeling rate of Tca8113 cells was as high as 93.18% after 6 h of co-culture. As the co-culture time increased, the cells continued to divide and the labeling rate was gradually decreased. Entrance of peptide conjugated QDs did not affect the proliferation, invasion, adherence and chemotaxis of Tca8113 cells. These results provided basis for the application of QDs in the non-invasive, *in situ* and visual studies of genesis, development and metastasis of tumor cells.

## 3. Experimental Section

### 3.1. Materials and Instruments

The human tongue squamous cell carcinoma cell line Tca8113 was provided by West China College of Stomatology, Sichuan University (Sichuan, China). Qtracker^TM^ 800 Cell Labeling Kit (Invitrogen, Carlsbad, CA, USA) uses a custom peptide to deliver QDs into the cytoplasm of live cells without cell-type specificity. Laser scanning confocal microscope was purchased from Leica (Tcs-sp5, Germany). Flow cytometry (BD Biosciences, San Jose, CA, USA) was used for detection of cell labeling. Cold low-speed centrifuge (Z233MK-2) was purchased from HERMILE (Germany). Transwell chamber (Corning Incorporated, Corning, NY, USA) was used for the chemotaxis experiment.

### 3.2. Labeling Tca8113 Cells with QDs

Labeling Tca8113 cells with QD800 was performed according to the manufacturer’s instructions. Briefly, trypsin-digested Tca8113 cells were transferred into 5 EP tube with 1 × 10^6^ cells in each tube and then 0.2 mL of QD800 labeling solution (10 nM) was added. After mixing, the cells were co-cultured with QD800 for 1 h and centrifuged (1,000 x *g*) for 5 min at 4 °C. The supernatant medium was discarded and the cell pellets were washed with PBS for twice to remove the uninternalized QDs. The QD800-labeled Tca8113 cells (Tca8113/QD800) in the 5 EP tubes were individually seeded in a 6-well plate for continuous culture. At 6 h, 1 d, 3 d, 5 d and 7 d post-labeling, the labeled cells were dispersed into suspension and the labeling efficiency was detected at these five time points. Laser-scanning confocal microscope was used to observe the distribution of QD within the cells after Tca8113 cells were labeled for 1 d. All the experiments were repeated for 3 times and the average of the labeling rate was calculated accordingly.

### 3.3. In Vitro Cell Growth Assay

Well-growing Tca8113 cells (2 × 10^4^ cells/mL) were inoculated into four 24-well plates with 0.18 mL in each well. After 24 h of culture, synchronous growth was achieved and these four plates were divided into four groups. To the control group was added with 0.02 mL of RPMI1640 medium containing 15% FBS. Experimental groups A, B and C were added with different amount of QD800 to achieve a final concentration of 5 nM, 10nM and 15 nM, respectively. Three wells of cells were randomly selected from each group every day and were completely digested with 0.1% of trypsin. Single cell suspension was obtained by pipetting and cell number was counted by using a hemocytometer for a consecutive eight days. All the experiments were repeated for three times and the average value was reported.

### 3.4. Plate Clone Formation Assay

Cell suspension (1 × 10^6^ cells/mL, 0.2 mL) was co-cultured with QD800 (10 nM) at 37 °C with 5% of CO_2_ for 1h, then centrifuged at 1,000 r/min for 5 min at 4° C to wash off the QDs that did not enter the cells for twice. The obtained Tca8113/QD800 cells were used for the following experiments immediately. Suspensions of Tca8113/QD800 or Tca8113 were seeded into two 60 mm Petri dishes with 2 × 10^2^ cells in each dish. The cells were dispersed evenly by slightly shaking the Petri dishes and then were incubated at 37 °C with 5% of CO_2_ for 21 d until the visible clones appeared. The medium was discarded and the cells were carefully washed with PBS for twice. After fixed with methanol for 15 min, the cells were stained with Giemsa's solution for 15 min before washing with tapping water and air-drying. The clonies with more than 50 cells were counted with an inverted microscope and the clone formation rate was calculated with the following formula: Plate clone formation efficiency = (number of clonies/number of cells inoculated) × 100%. All the experiments were repeated for 3 times and the average values were reported.

### 3.5. Detection of Cell Adherence

Cell suspension (5 × 10^6^ cells/mL) of Tca8113/QD800 were prepared according to the procedure described above. The obtained Tca8113/QD800 cells were used for the following experiments immediately. Cell suspension (5 ×10^6^ cells/mL) was prepared for both Tca8113/QD800 and Tca8113 and was seeded in 24-well plates with 0.2 mL in each well and 12 wells for each experimental group. After 30 min, 60 min, 90 min and 120 min of incubation, three wells of cells in each group were washed with PBS to remove non-adherent cells and the adherent cells were digested with 0.25% of trypsin for cell counting using a microscope. The cell adherent rate was calculated using the following formula: cell adherent rate = (the number of adherent cells/total number of cells) × 100%. All the experiments were repeated for three times and the average values were reported.

### 3.6. Detection of Cell Invasion

Tca8113/QD800 cells that were prepared according to the procedure described above were used for the following experiments immediately. Cell invasion and reconstruction of basement membrane experiment was performed in Transwell that was separated into upper and lower chambers by a poly carbonate acid filter membrane containing multiple 8 μm-diameter pores. Fifteen micrograms of matrigel were poured in the upper and lower sides of the filter membrane for 1 h of reconstruction at 37 °C before uses. Tca8113/QD800 or Tca8113 cells (2 × 10^5^) were added into the upper chamber and incubated at 37 °C for 12 h. The cells that invaded into the lower side of the membrane were stained with HE solution and five fields of views (400 × magnitude) were randomly selected and the cell number was counted. All the experiments were repeated for three times and the average number was reported to indicate the invasive ability of Tca8113 or Tca8113/QD800 cells.

### 3.7. Cell Chemotaxis Assay

In cell chemotaxis experiment, the matrigel was not applied on the surface of the filter membrane. All the other procedures were identical to the cell invasion assay. All the experiments were repeated for 3 times and the average number of cells in the lower side of the filter membrane was reported to indicate the chemotactic ability of Tca8113 or Tca8113/QD800 cells.

## 4. Conclusions

Non-invasive cancer imaging and tracer studies based on the use of near-infrared fluorescent quantum-dot inside the cells have shown significant prospects for development [7,10–12], but the primary key information needed to determine if using quantum dots labeled cancer cells to undertake non-invasive imaging study is scientific is whether labeling of cancer cells by a large number of QDs will affect their proliferation, invasion and metastasis. To date we have not seen any relevant reports in this regard. Our study used peptide-conjugated near-infrared fluorescent quantum dots for labeling Tca8113 cancer cells, and colony forming experiments with flat-panel, transwell chamber invasion and scouring experimental methods. These experiments proved that QD800 labelling of Tca8113 cancer cells does not affect their growth and proliferation, invasion and metastasis, and offered a scientific basis for *in vivo* non-invasive cancer imaging and tracer studies using near-infrared fluorescent quantum dots.

## Figures and Tables

**Table 1.** Adhesion of Tca8113/QD800 and Tca8113 cells at 30, 60, 90 and 120 min post labeling.

**Time(min)**	**Adhesion rate (%)**		***P* value(χ^2^ test)**
	**Tca8113/QD800**	**Tca8113**	
30	10.3 ± 1.5	10.7 ± 1.6	>0.05
60	16.5 ± 2.4	15.9 ± 2.8	>0.05
90	27.2 ± 3.4	30.1 ± 2.9	>0.05
120	39.5 ± 3.6	40.8 ± 3.8	>0.05
